# Alien Attack: A Non-Pharmaceutical Complement for ADHD Treatment

**DOI:** 10.3390/e23101321

**Published:** 2021-10-11

**Authors:** Sofia Ahufinger, Pilar Herrero-Martín

**Affiliations:** Center of Biomedical Technology (CTB), Universidad Politécnica de Madrid (UPM), 28223 Pozuelo de Alarcón, Spain; s.ahufinger@alumnos.upm.es

**Keywords:** attention-deficit hyperactivity disorder (ADHD), executive functions (EFs), awareness of interaction, human computer interaction (HCI), user-centered design, software engineering

## Abstract

Mental health issues are among the most common health issues nowadays, with attention-deficit hyperactivity disorder (ADHD) being the most common neurobehavioral disorder affecting children and adolescents. ADHD is a heterogeneous disease affecting patients in various cognitive domains that play a key role in daily life, academic development, and social abilities. Furthermore, ADHD affects not only patients but also their families and their whole environment. Although the main treatment is based on pharmacotherapy, combined therapies including cognitive training and psychological therapy are often recommended. In this paper, we propose a user-centered application called Alien Attack for cognitive training of children with ADHD, based on working memory, inhibitory control, and reaction-time tasks, to be used as a non-pharmacological complement for ADHD treatment in order to potentiate the patients’ executive functions (EFs) and promote some beneficial effects of therapy.

## 1. Introduction

Mental health is one of the main issues in our society nowadays, especially attention-deficit hyperactivity disorder (ADHD), which is characterized by a persistent pattern of inattention and/or hyperactivity and impulsivity [1,2] and is usually diagnosed before the age of 12. It is the most commonly diagnosed childhood neurobehavioral disorder. Patients with ADHD present impairments across various domains, such as difficulty paying attention and staying focused, together with motivation deficits. Children with ADHD also have difficulties organizing their time and assignments, as well as finishing school tasks and homework or even socializing [3,4,5], which can lead to significant academic problems, poor quality of life and the extension of the ADHD into the later stages of life [6,7,8]. Furthermore, ADHD is a heterogeneous disorder affecting each patient in a different way [9], and it affects not only patients but also their families and their whole environment [10]

At present, there is no cure for ADHD; the main treatment is based on daily stimulant (i.e., methylphenidate) pharmacological therapy focused on reducing the main symptoms. Nevertheless, although stimulant therapy shows the largest positive effects in mitigating symptoms, a significant proportion of diagnosed patients do not respond to the treatment, mostly due to the heterogenicity of this disease. Additionally, pharmacotherapy can have both physical and psychological side effects [11]. For this reason, pharmacotherapy is often used in combination with various psychological approaches such as cognitive training, to potentiate the effect of the treatment. Cognitive training, as applied to ADHD, aims for the development of new cognitive skills, and it is based on neural plasticity, taking account of studies performed on dementia and brain-injury rehabilitation [12,13,14].

ADHD has also been associated with hyperfocus (the experience of deep and intense concentration for hours) [15] when the patient’s motivation is high. Various studies demonstrate the positive impact of serious games on improving personal key skills among children with learning difficulties [2,16,17]. In fact, Zheng et al. classified serious games relevant to ADHD according to different platforms and conducted a systematic review of video games that can help children with ADHD [18].

In this paper, we propose a user-centered application called Alien Attack, composed of different minigames for cognitive training of children with ADHD that motivate children to potentiate their executive functions (EFs) [19] by means of positive reinforcement and promote some beneficial effects of therapy [20,21].

## 2. Designing a Serious Game for ADHD

Nowadays more than ever, technological and digital approaches are being applied to various fields, including healthcare. Video games have been used for decades as entertainment. However, during recent years, there has been an increasing interest in the use of games for teaching and training or even for treating various diseases [22]. Games used for these types of purposes, i.e., learning, training, or the treatment of a particular disease, rather than just for entertainment, are called “serious games” [17]. Game elements increase the user’s engagement in the proposed activities, and it is well known that learning by playing leads to faster and more internalized learning [23].

The specific application of these digital technologies to mental healthcare is known as “e-mental health” [24]. One of the groups of patients in this field that might potentially benefit from the use of serious games are children and adolescents with ADHD [17]. The main advantage of these types of games is their capacity for engaging and interesting patients by balancing the various goals that they must achieve with their own skills [25], allowing them to see the intervention as a fun activity instead of a tedious task that looks like homework [26,27]. This potential for increasing the patients’ engagement and motivation, as well as encouraging them to stay focused on specific tasks, can also lead to an increase in the effectiveness of neurocognitive training in patients and improved general outcomes [28].

Serious games for ADHD (SGADs) can help with ADHD diagnosis and treatment in children, and currently there are many SGADs that have been developed for mobile platforms, so that patients with ADHD can be treated anytime and anywhere [17]. When using SGADs to treat patients with ADHD, the patients’ impulses can be suppressed and attention, life skills and social skills can be improved. Furthermore, using serious games as an auxiliary and complementary tool can not only alleviate the symptoms of ADHD patients but also improve their executive functions and be used to conduct cognitive training [29,30].

SGADs can be divided into three categories, according to different game platforms: console games, computer games, and mobile games [17]. Mobile games allow patients with ADHD to be treated anytime and anywhere, which is an important advantage when these patients are children, as they can be treated without even being conscious of their therapy, allowing ADHD patients to actively participate in the treatment process and thereby completing the training process smoothly and effectively.

Considering previous studies that prove the benefits of using SGADs for reducing the symptoms of children affected by ADHD and improving their executive functions [17,31], we decided to create a positive-reinforcement serious game [32] focused on cognitive training through a process-based approach, based on repeating different cognitive tasks belonging to various cognitive domains. The game, called Alien Attack, was designed using a user-centered design process to create an attractive, easy-to-use, and efficient SGAD.

### 2.1. Alien Attack User-Centered Design

The user-centered design approach actively involves end users during the whole process. Thus, it is important to know and understand both the users and the tasks, as well as the environment in which the tasks will be performed. The framework presented in the ISO 9241-210:2010 standard is a framework for this type of design that tries to guarantee the accomplishment of all goals and requirements by following an iterative process.

It is important to start by planning the design process, to define the project goals and requirements, based on the current state of the art and the advice of the experts consulted. Once the goals of the project are defined, the first step of the user-centered design approach is to understand and specify the context of use, which includes the characteristics of the end users, the equipment, and the physical and social environment in which the system will be used, as well as the tasks that users need to perform, how they are performed, the time required and any difficulties that might be encountered.

Although the profiles of the target groups might share some characteristics, each of the targeted groups must have a specifically defined context of use. To define this context of use, observations and interviews with users are performed. Two kinds of observations are made: ethnographic observations, which involve observing and analyzing users without interacting with them and contextual observations, where the users are also interviewed.

In this case, the group of participants included children from 6 to 13 years old from two schools (Antanes School and Legamar School), located in the south of Madrid. Two kinds of observations were performed. Firstly, children were observed and analyzed without interacting with them, in various places such as their houses or the hospital waiting room, thus performing an ethnographic observation. Secondly, contextual observations were made. In both cases, the tasks consisted of observing while they played mobile games.

After the observation process, the users were interviewed to obtain information that was important for understanding the context of use as well as the user preferences. In this case, this information allowed us to obtain a clear idea of the children’s preferred types of games and their favorite games throughout their lives, their preferred characterization of avatars, and the preferred types of interactions and types of environments in which avatars move. This information was essential for starting to design the minigames.

Using the obtained information, the context of use can be understood, leading to the specification of the user requirements (as seen in this paper), which takes the process to the next phase of generating design solutions that attempt to satisfy all the requirements and the children’s needs.

The second phase of the framework involves creating different design solutions based on the defined context of use. For this purpose, we created low- and high-fidelity prototypes, which are limited representations of the system, paper based in the case of the low-fidelity prototype and computer based in the case of the high-fidelity prototype, thus allowing quick modifications and improvements.

The user-centered design process includes various validation points, where prototypes are evaluated by end users via usability tests. The results obtained from these tests demonstrate whether the context of use and the requirements are correctly defined and offer important information about the design solutions, detecting possible mistakes and suggesting improvements in some cases.

**Figure 1:**
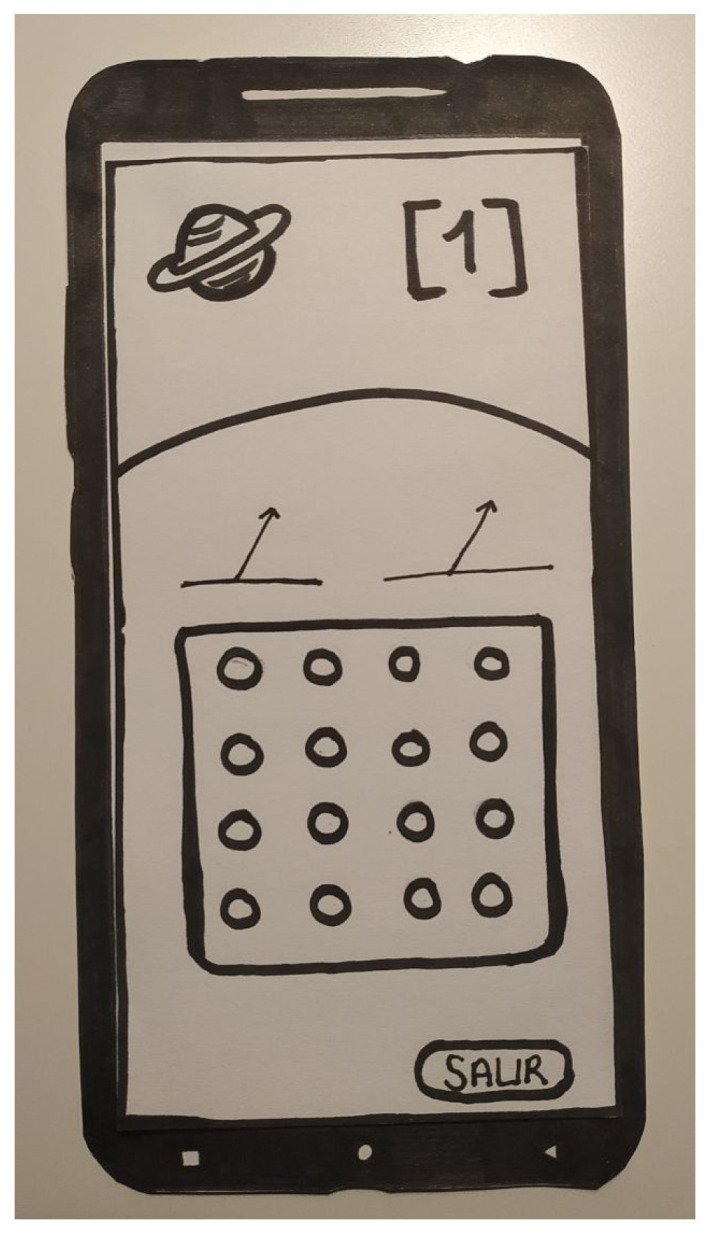
Low-fidelity prototype of Alien Attack, on paper, showing one of the minigames.

In our case, the first evaluation was performed using a low-fidelity paper prototype (Figure 1). The participants were asked to perform some tasks in various minigames, following the think-aloud protocol. In this evaluation, we detected that the initial design presented by the prototypes used language that was far removed from the language used and understood by the children (our target group). We therefore had to modify the interaction messages to avoid some expressions and words. We also had to modify the locations of several items, to prevent the children making mistakes. The detection of these types of issues using the low-fidelity paper prototype allowed quick correction of the design before the final implementation of the system.

**Figure 2:**
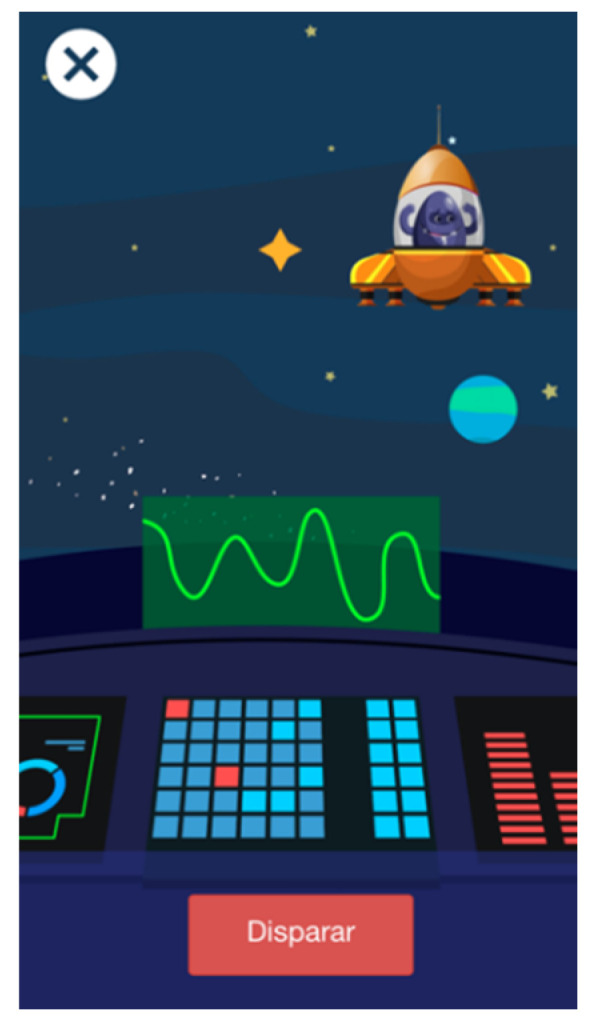
High-fidelity prototype of Alien Attack showing one of the minigames.

The second evaluation was performed with the high-fidelity prototype (Figure 2), which was built with the open-source Quant-UX tool (https://quant-ux.com (accessed on 4 October 2021). In the high-level prototype, each of the minigames had an introductory text at the beginning explaining how to play and therefore how to carry out the task. Some children skipped this part, and when they were ready to play they were not sure what to do. For this reason, we decided that the brief introduction of each of these minigames should not only be a written text but should also include an audio explanation. In addition, some of the pointers were ignored, so it was clear that some important information was missing. In conclusion, the high-fidelity prototype suggested modifications to the method of interaction with the final users.

The prototypes were iteratively evaluated by the users to determine, on the one hand, whether the context of use had been correctly understood and specified and, on the other hand, whether the user requirements had been correctly specified. The evaluation of the prototypes took place in a controlled environment (the child’s house). In this case, the participants were students within the same age range from the aforementioned schools. This process was repeated several times until all the detected issues were related to the requirements. The process required approval from an ethics committee, in order to carry out the research and its evaluation.

Finally, once the design modifications and corrections are performed, the next step involves implementing the system (in our case, Alien Attack) and moving forward to the next validation point, which evaluates the implemented solution. This type of validation uses the end user’s point of view, as previously, to develop higher stability and greater confidence in the system performance. These tests are often called “black-box” tests, due to the fact that testers (end users) have no information on the source code or other details of the implementation. On the contrary, testers are only concerned about the behavior of the system when performing the defined test activities. Each function of the system is specifically tested by providing the appropriate input and evaluating the obtained output with respect to the expected results. The aspects of the system mainly evaluated, besides the mainline functions and possible error conditions, were its basic usability and the main principles of the framework previously evaluated using the low- and high-fidelity prototypes. The results obtained in the evaluation of the implementation of Alien Attack are explained in a subsequent section.

## 3. Implementing Alien Attack

We designed a cognitive training game for schoolchildren with ADHD to improve their executive functions (EFs), following a user-centered design process. Based on the executive dysfunction framework as well as on learning theory, we selected some tasks from widely used measures of school EF to be gamified, creating a minigame for each one of the selected tasks.

We chose to focus on working memory (WM) [33] and inhibitory control (IC). For WM, which has been shown to be potentially benefited by this training [34], we decided to include the n-back task [35] and the Corsi task [36] for visuo-spatial memory training. For IC training, we included the Eriksen flanker task (interference control) [37] and the go/no-go task (response inhibition) [38]. Finally, for reaction time, we included the Deary–Liewald task [39] and the Simon task [40]. The following sections explain the various minigames designed and the mechanisms of each game.

### 3.1. Minigame 1: Corsi Task

**Figure 3:**
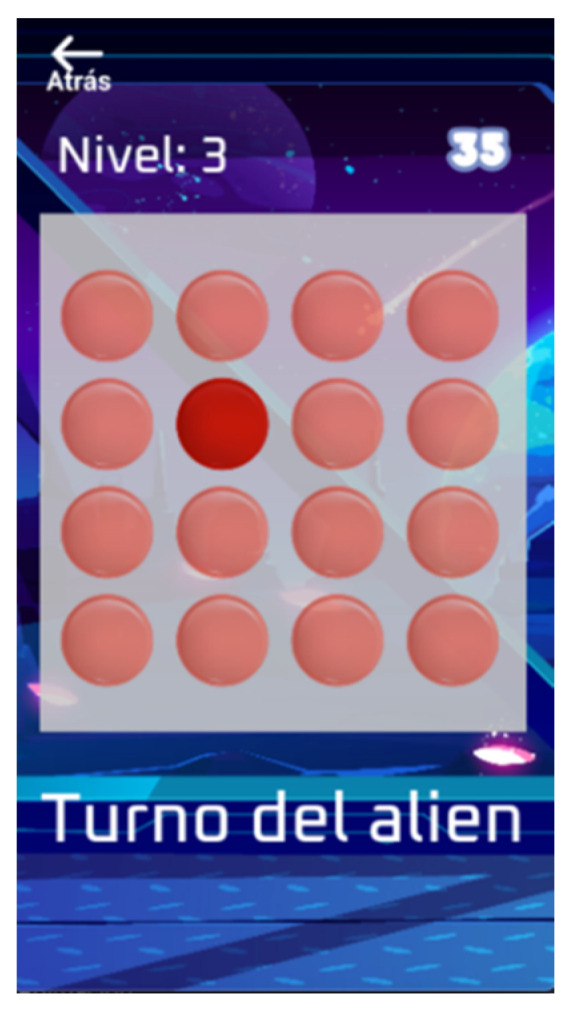
Gamification of the pattern span task. In each turn, the alien presents a sequence that the player must replay during the turn.

This minigame is based on visuo-spatial memory training and presents the player with a matrix of dots in which a sequence of dots is illuminated (Figure 3). The player must pay attention and remember the sequence of illuminated dots (in order) and repeat it afterwards, which requires memory for positional sequences. When the player repeats the sequence accurately, the difficulty of the game increases by making the sequence one illuminated dot longer. If the player fails when repeating a certain sequence, the sequence is repeated. If the player fails the same sequence twice in a row, then the game finishes.

### 3.2. Minigame 2: Flanker Task

**Figure 4:**
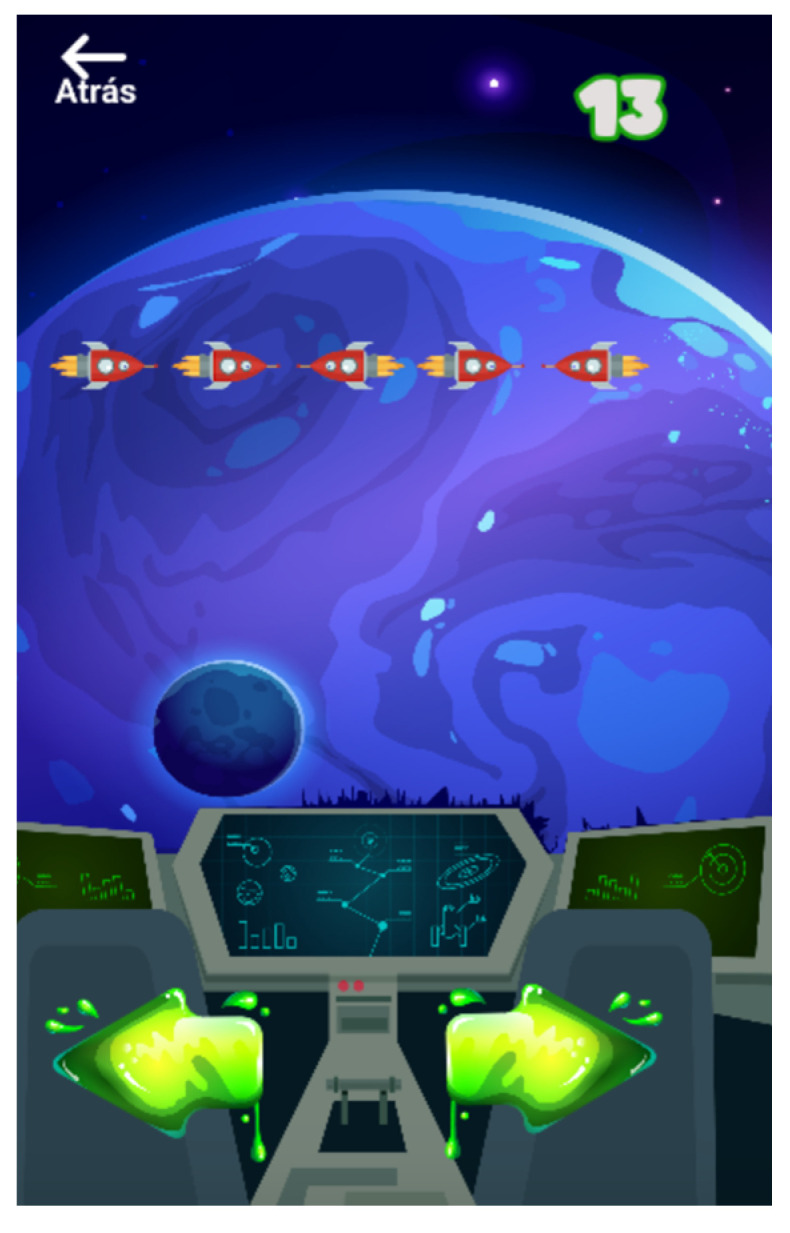
Gamification of the flanker task. A row of rockets is presented, and the player is asked to determine the direction of the central stimulus (rocket).

The flanker task (Figure 4) is widely used for inhibitory control training. In this minigame, a row of five stimuli, in this case rockets, is presented to the player, who is asked to indicate the direction of the central stimulus (left or right). When all the rockets are facing in the same direction it is called a “congruent” trial, and when the central stimulus is facing in the opposite direction it is called an “incongruent” trial. The player must complete 2 blocks of 15 trials, where half are congruent trials and half are incongruent trials in each block. Each row of rockets is presented for 2000 ms and the difficulty increases by reducing the duration of appearance of the row on the screen if the player achieves a correct response to 75% of the trials in the first block. The game lasts until the two blocks are completed or until the player fails twice in a row.

### 3.3. Minigame 3: N-Back Task

**Figure 5:**
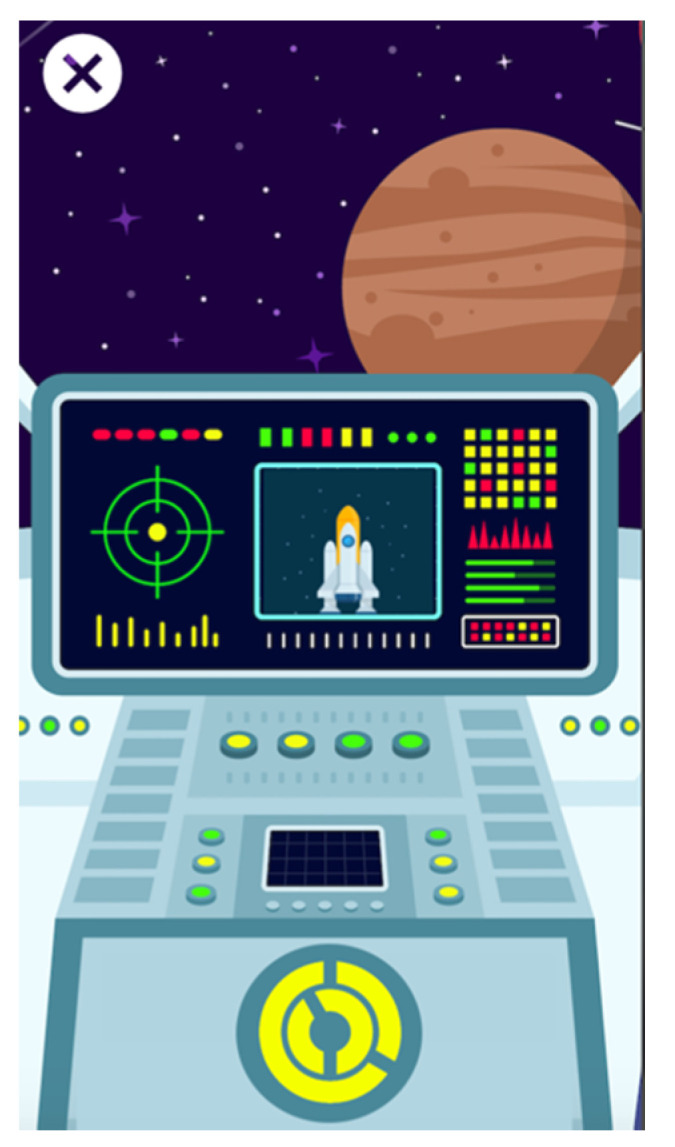
Gamification of the go/no-go task. Images of allied and enemy spaceships are presented, and the player is asked to shoot only the enemy spaceships.

This minigame (Figure 5) is based on the n-back task, which is used to train working memory (WM). The player is shown a series of images of spaceships and is told to touch the screen only when the current spaceship is the same as the n-back spaceship image. The first level is the 1-back level, so the player must touch the screen only if the current spaceship is the same as the previously presented spaceship. This minigame is composed of 2 blocks of 15 trials, with one third of the trials being hit trials (images which require the player to touch the screen) in each block. The first block is a 1-back level, and if the player achieves a correct response in 75% of the hit trials, the second block is a 2-back level (where the player must touch the screen only if the current spaceship is the same as the spaceship before last). Spaceships are shown for 2000 ms and the difficulty is increased by increasing the time between stimuli by 1000 ms, from 1000 ms to a maximum of 3000 ms. The game lasts until the two blocks are completed or until the player fails twice in a row.

### 3.4. Minigame 4: Go/No-Go Task

**Figure 6:**
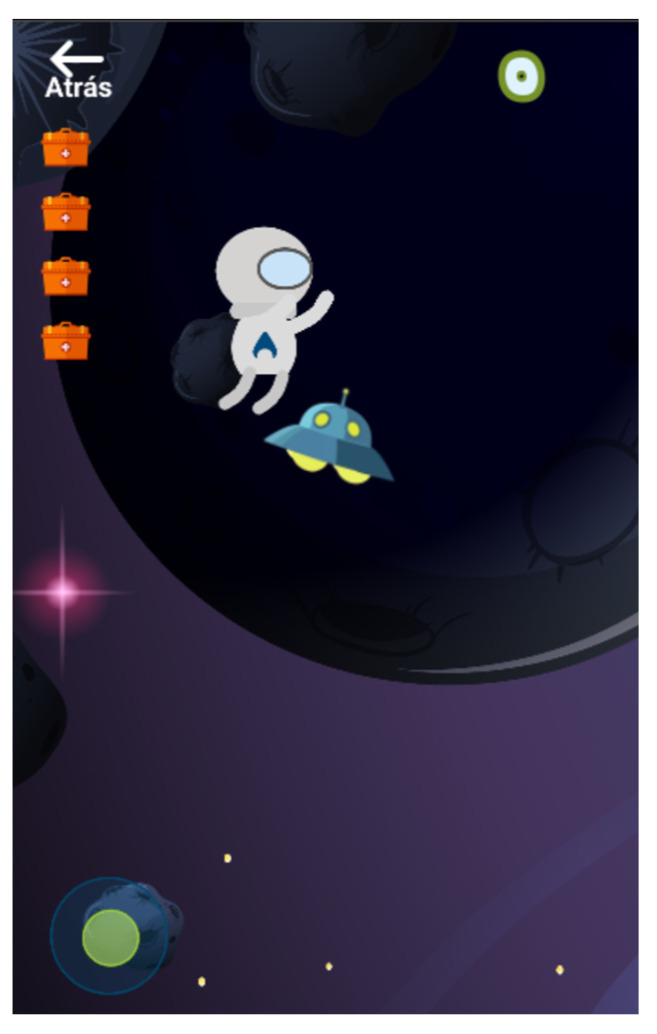
Gamification of the Deary–Liewald task created for the Alien Attack game. This minigame focuses on reaction time, asking the user to shoot the alien as quickly as possible.

This minigame (Figure 6) focuses on inhibitory control training and asks the player to shoot the alien (enemy) spaceships while avoiding shooting the allied spaceships. The player must complete 2 blocks of 15 trials with a ratio of two go stimuli to each no-go stimulus. Each stimulus (spaceship) is presented for 2000 ms, but if the player achieves a correct response in 75% of the no-go trials, the stimuli are presented for 1200 ms during the second block. The game lasts until the two blocks are completed or until the player fails twice in a row.

### 3.5. Minigame 5: Deary–Liewald Task

The Deary–Liewald task involves simple reaction time, which involves making a response as quickly as possible when a single stimulus is presented, and choice reaction time, which is more complicated and involves making an appropriate response to only one of the presented stimuli. In this case, the game (Figure 6) is composed of two parts. In the first part, the player is asked to pay attention to a presented window and press the button as fast as he/she can whenever an alien appears. The player must complete 2 blocks of 15 trials in this first phase. The stimulus (alien) appears at a randomly selected frequency (time between stimuli) from 1000 to 3000 ms. The second phase is the same as the previous one but presents four windows and four buttons instead of only one. The alien appears each time in one randomly selected window and the player must press the corresponding button. The game lasts until the two blocks of both phases are completed.

### 3.6. Minigame 6: Simon Task

**Figure 7:**
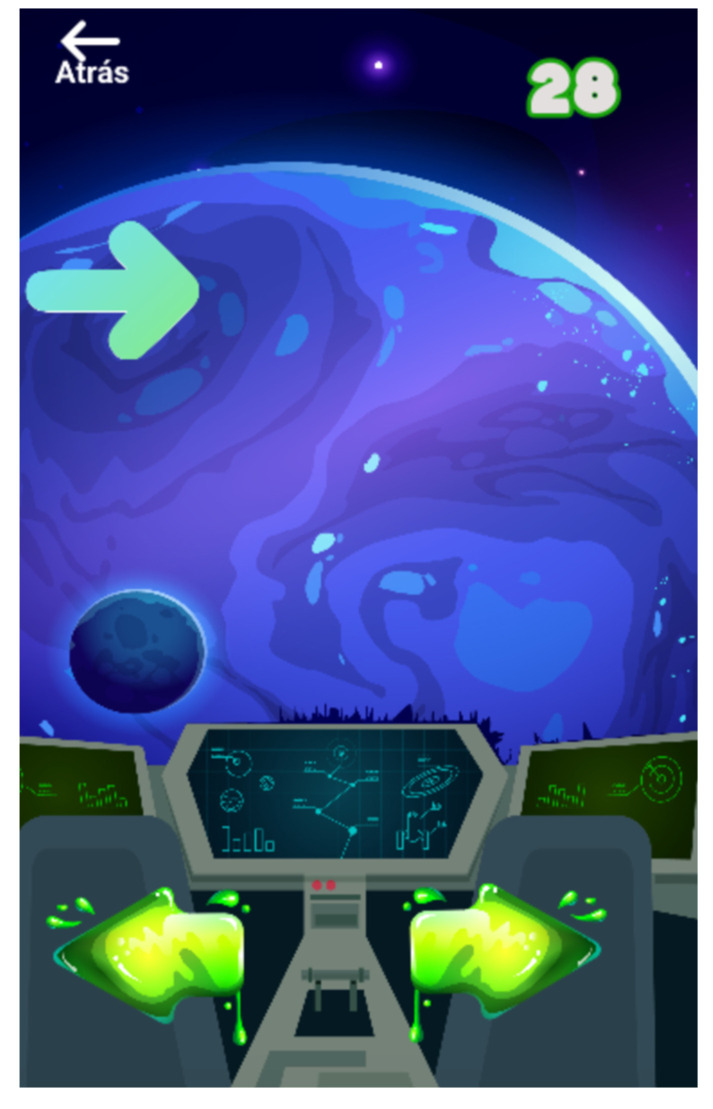
Gamification of the Simon task. The arrows appear on different sides of the screen and the player is asked to determine the correct direction of the presented arrow.

This minigame (Figure 7) focuses on reaction time and asks the player to determine the direction of a presented arrow, irrespective of the side of the screen on which the arrow appears, while piloting a spaceship. The stimulus (arrow) is presented for 1500 ms with a time between stimuli of 2000 ms. The game is composed of 2 blocks of 15 trials each and it ends either when both blocks of trials are finished or when the player fails twice in a row.

When each minigame is finished, the player receives a number of colored coins, based on the score obtained in each game. With these coins, the player can perform different repairs on the spaceships and buy new accessories for the main character. The spaceship repair shop screen provides information about the number of coins the player has, the available repairs, and the state of the spaceship (which can be translated into the level and performance of the player in the game), as well as the available accessories.

### 3.7. UML Diagrams and Task Activity Diagrams

**Figure 8:**
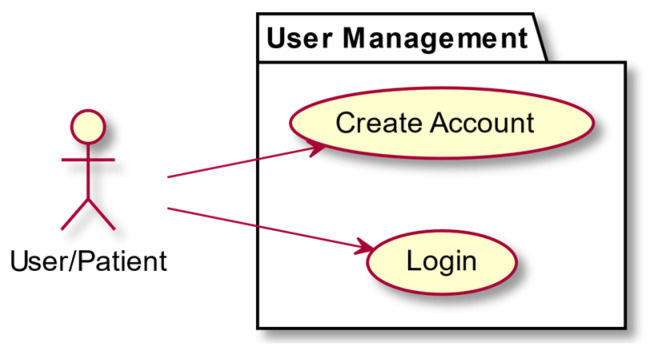
User management.

**Figure 9:**
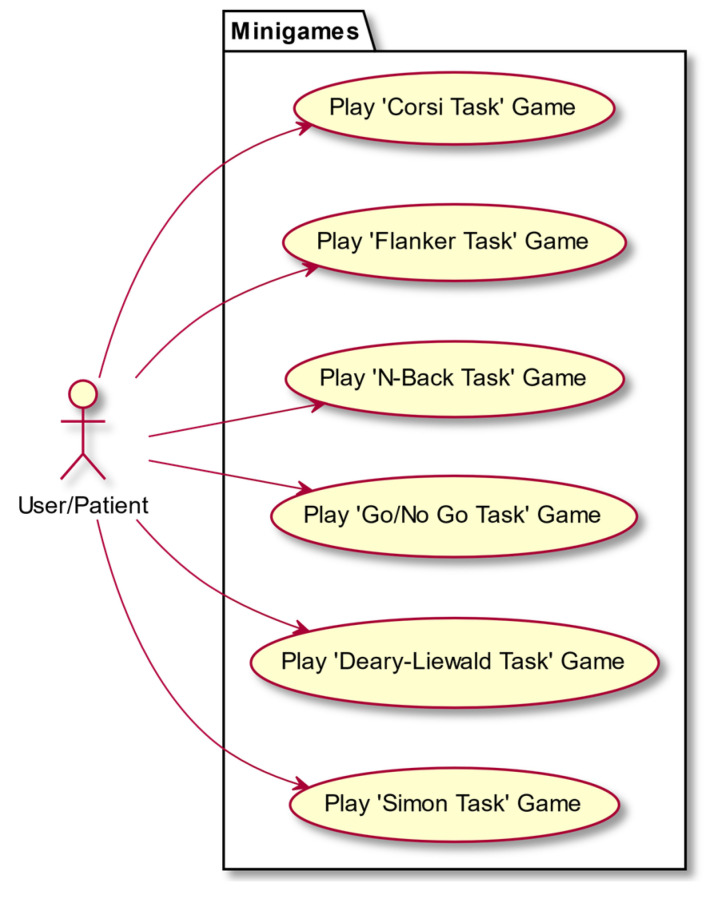
Minigames.

**Figure 10:**
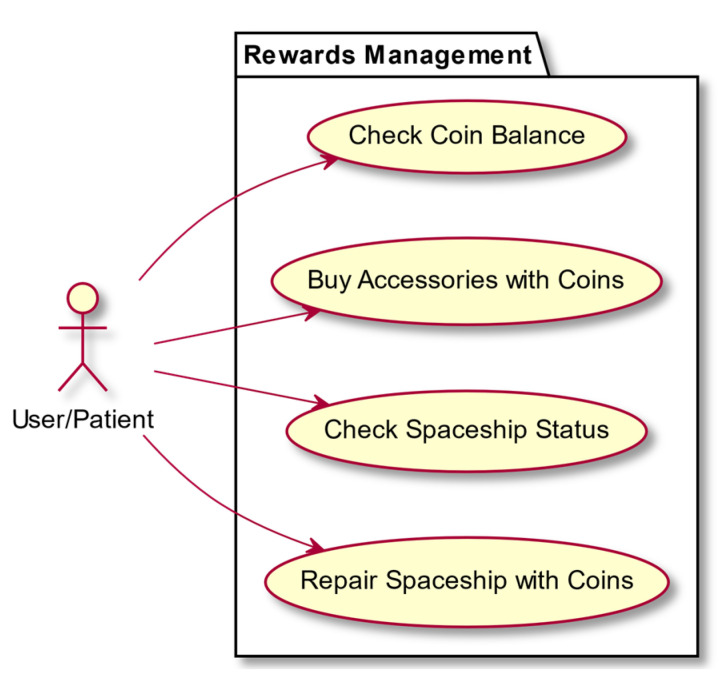
Rewards management.

**Figure 11:**
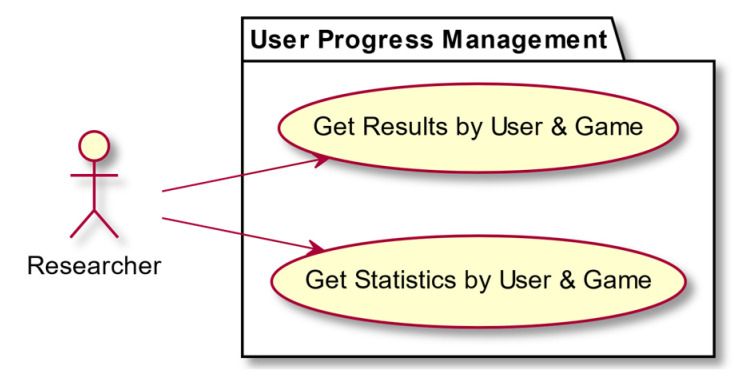
User progress management.

**Figure 12:**
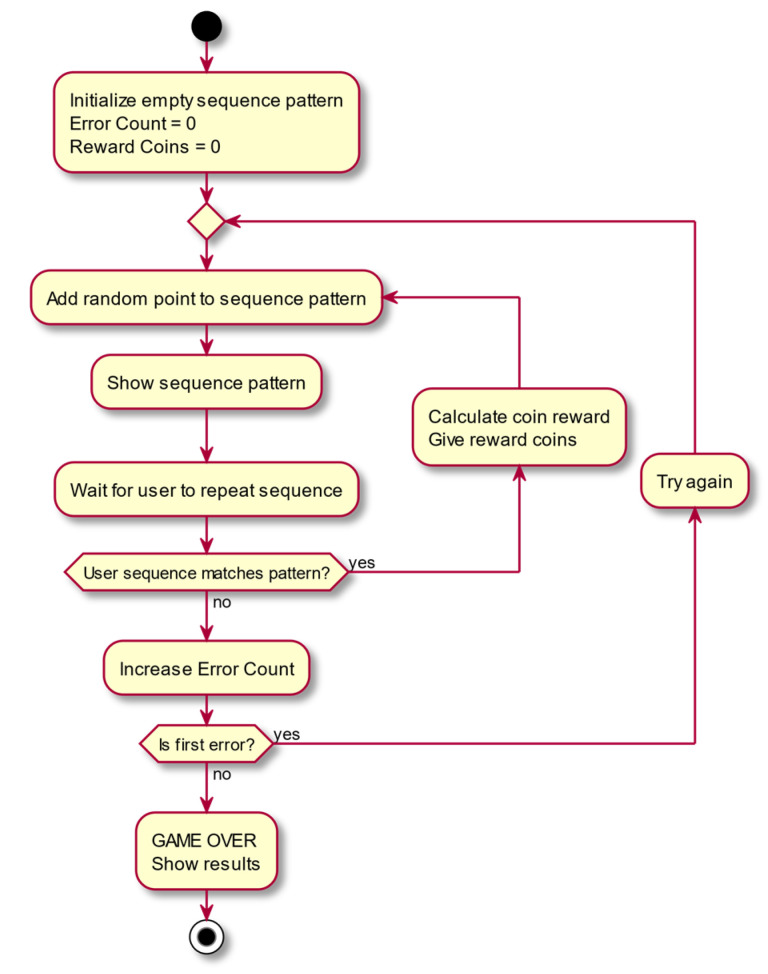
Corsi task activity diagram.

In this section we present some use-case diagrams describing the high-level functions and interactions between the system and its actors (Figure 8, Figure 9, Figure 10 and Figure 11), as well as the task activity diagram for the first minigame, Minigame 1, the Corsi task of the light sequences (Figure 12).

## 4. Alien Attack Validation

Alien Attack mainly focuses on two objectives. The first is the straightforwardness of the interaction mechanism in each phase of the game, resulting from the user-centered design, which emphasizes this aspect due to the needs of the final users and their skills. The second concentrates on improving the users’ executive functions (EFs). The functional testing attempts to verify whether or not the system behaves in conformance with the specified requirements and needs, considering the aims for which the software was designed.

As mentioned in Section 2.1, we followed the complete user-centered design process according to ISO 9241-210:2010, starting with the observations and interviews, followed by the specification of the context of use and the requirements, and finally, evaluating both the low- and high-fidelity prototypes, together with the SUS questionnaire. This process requires approval from an ethics committee in order to carry out the research and its evaluation.

Low- and high-fidelity prototypes were iteratively evaluated by the users to determine, on the one hand, whether the context of use had been correctly understood and specified and, on the other hand, whether the user requirements had been correctly specified. This evaluation is part of the user-centered design process, and for this reason, this process was discussed in Section 2.1. In this section, we focus on the analysis of the SUS questionnaire, as well as some other aspects that we consider important in the context of this article, such as the time required to perform each of the minigames, or the maximum level reached by our users.

The validation was carried out in different phases due to the socio-sanitary situation that we are experiencing as a result of the COVID-19 pandemic. When we carried out the first round of validation we were in a high-risk situation, and this validation had to be performed online. The second round of validation was face to face, but in the children’s homes, and the third also took place in their homes, during the period between the beginning of their holidays and their return to school. In total, 20 children aged 6 to 13 years old were evaluated, and 10 of them had ADHD while the other 10 did not. All of them were studying at Antanes School or Legamar School, both located in the south of Madrid.

We strongly believe that performing the validation test at home, in the real context of use, helped the children to feel more comfortable and involved with the tasks, providing more realistic feedback, since children are often apprehensive or nervous when taken to usability laboratories to perform this type of testing.

First, the children were asked to answer some short questions, and then they were asked to perform the following tasks within a given time:

1. Enter the second minigame and play up to level 5 (or up to the point of failure).
2. Enter the third minigame and play up to the point of failure.
3. Enter the fourth minigame and play up to the point of failure.
4. Enter the first minigame and play to the end of all the lives.

All the participants correctly finished the tasks before reaching the time out. Furthermore, some participants suggested that they would like an increase in the difficulty of the minigames.

After performing all the tasks, the participants were asked to answer an SUS questionnaire with the help of their parents, giving their opinions about the best/worst parts of the game and any other suggestions. The SUS questionnaire consisted of 10 short questions which should be scored using a scale from 1 (strongly disagree) to 5 (strongly agree). The final score of the questionnaire provided easy and reliable information about how people perceived the usability of the game [41].

Table 1 and Table 2 show the SUS scores for each of the participants, including the mean and the confidence interval, allowing the evaluation of the application’s usability.

Most of our results were in the higher range (90th percentile), which is considered excellent.

Additionally, the NPS^®^ (net promoter score) evaluates the probability of a user sharing the application in the user’s own environment. The range of the scale goes from −100 (no users would share the application) to 100 (all users would share it). The NPS^®^ score can be estimated using the SUS score, in this case giving an NPS^®^ score of 70%, showing that it is highly likely that our users would like to share our application.

Although the mean SUS score was quite similar for children with and without ADHD, we also considered that, in the context of this article, the performance time for each of the minigames and the maximum level reached by our users were both important (as shown in Figure 13, Figure 14, Figure 15, Figure 16, Figure 17, Figure 18, Figure 19, Figure 20, Figure 21 and Figure 22).

**Figure 13:**
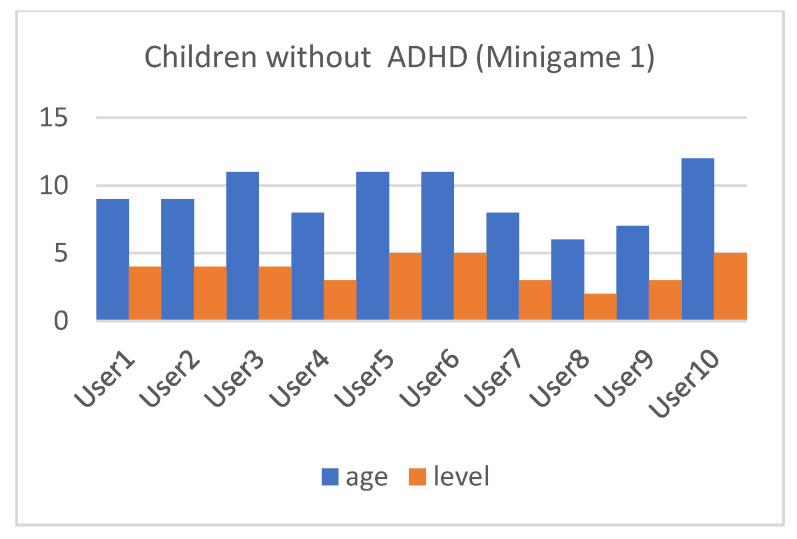
Minigame 1: results for children without ADHD.

**Figure 14:**
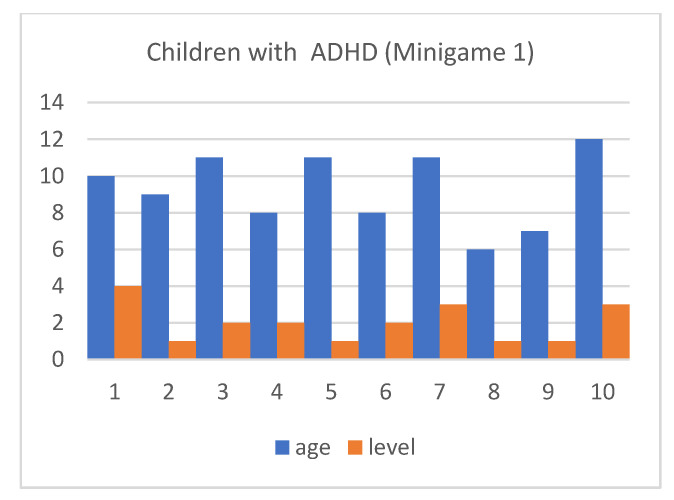
Minigame 1: results for children with ADHD.

**Figure 15:**
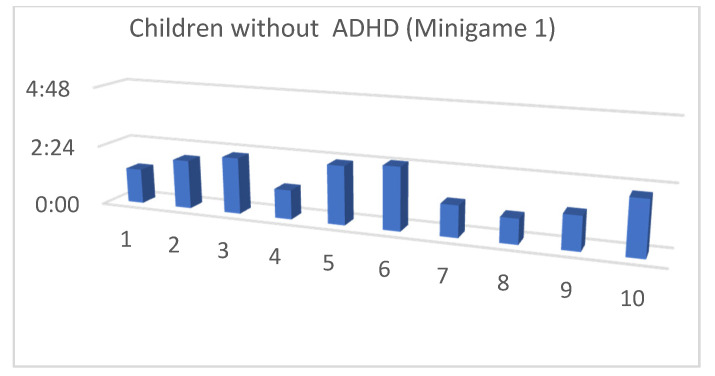
Minigame 1: time to finish the task.

**Figure 16:**
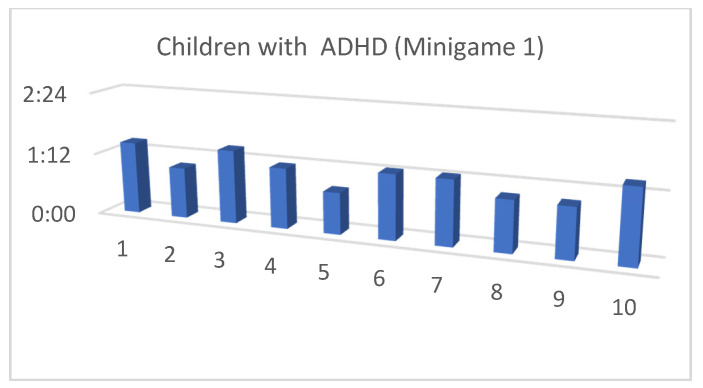
Minigame 1: time to finish the task.

**Figure 17:**
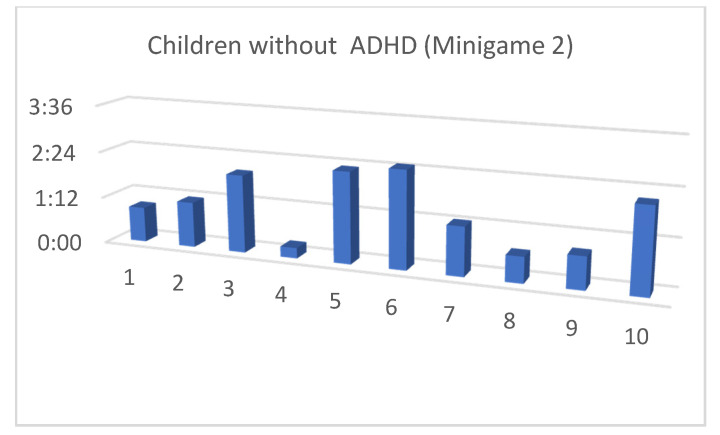
Minigame 2: time to finish the task.

**Figure 18:**
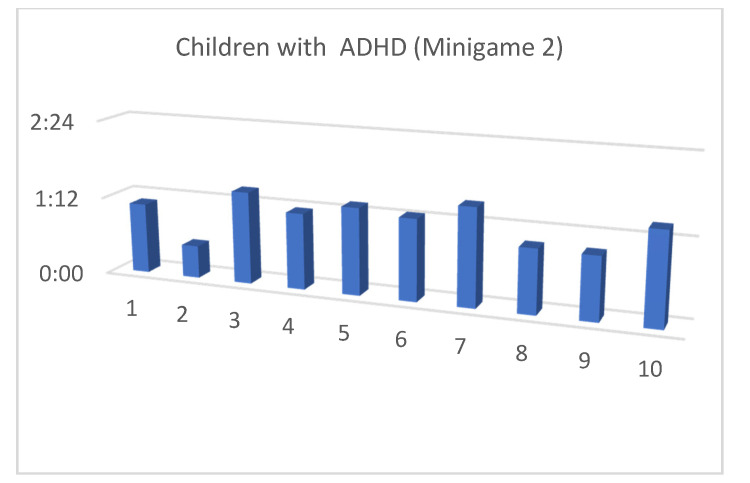
Minigame 2: time to finish the task.

**Figure 19:**
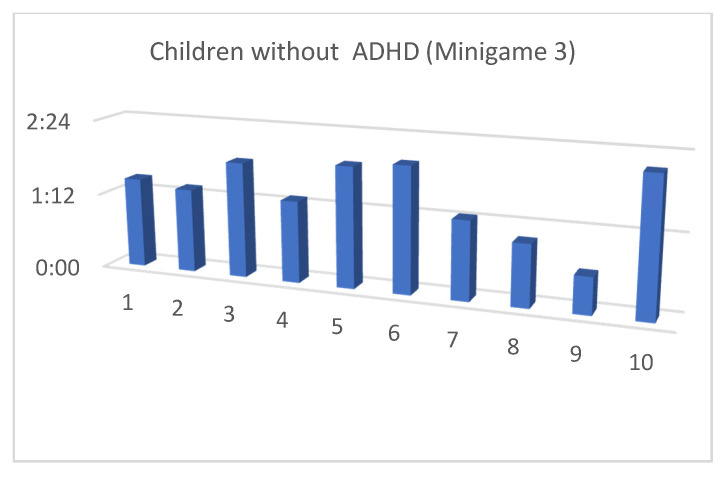
Minigame 3: time to finish the task.

**Figure 20:**
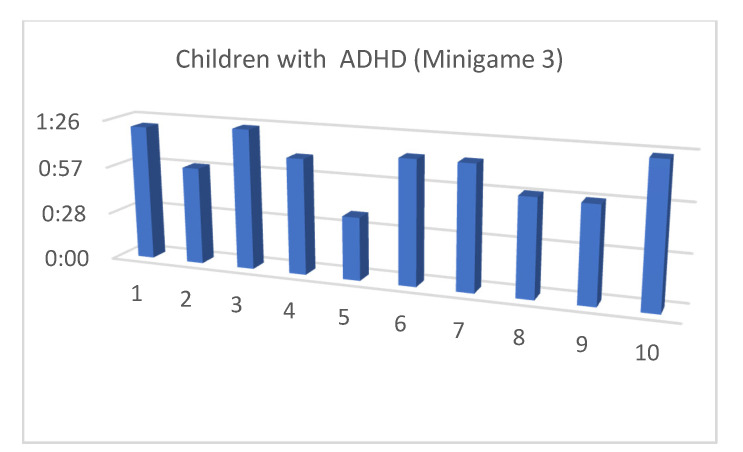
Minigame 3: time to finish the task.

**Figure 21:**
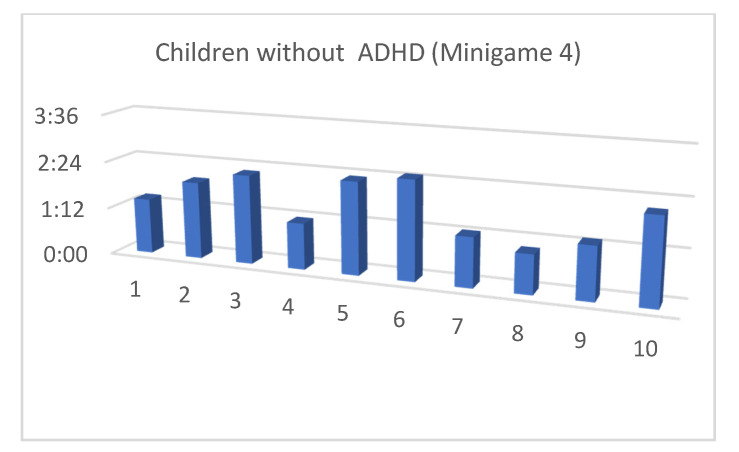
Minigame 4: time to finish the task.

**Figure 22:**
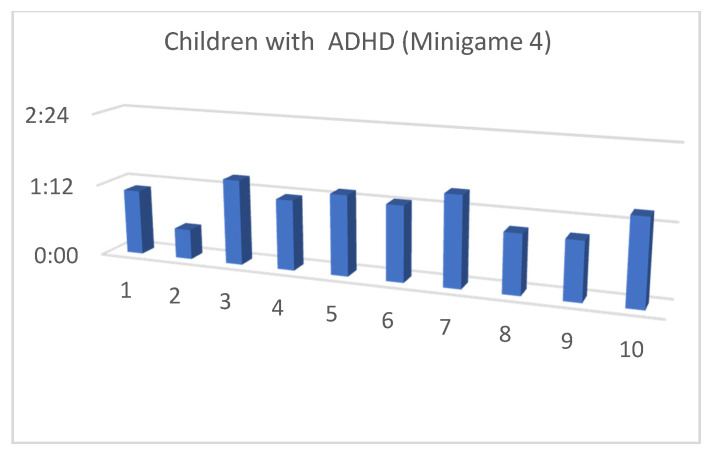
Minigame 4: time to finish the task.

Considering the previous figures (Figure 13, Figure 14, Figure 15, Figure 16, Figure 17, Figure 18, Figure 19, Figure 20, Figure 21 and Figure 22), we can observe that, in general, the time required to finish each task was somewhat less in the case of children with ADHD, but in some cases the children were very excited and somewhat nervous, knowing that they were playing Alien Attack for the first time (a new game that none of their friends knew yet) and they completed the tasks very quickly.

User satisfaction was high: in fact, at the end of the evaluation, some children wanted to try again and some of them asked for new and more complicated levels/minigames.

The results show that the system is consistent and intuitive, since none of the testers declared any problem with using the system, and 100% of users stated that they were able to use the system without previous knowledge and felt engaged and comfortable with it. All of them enjoyed the game and none thought that new users would need help to use the game for the first time. In summary, the children enjoyed playing the game and would have liked to continue playing. The main suggestions received were to include higher levels with increased difficulty and to include an online gaming mode.

## 5. Conclusions

This application could offer a potential non-pharmaceutical complement for the treatment of ADHD, since it is well known that games optimize motivation and treatment effects in these children. These types of technologies allow patients to remain at home during treatment, increasing their engagement, comfort, and satisfaction, which might potentiate the effect of the pharmacological therapy [16,17,42].

Due to the good results and the feedback obtained during the validation of the system, we consider our system to be highly consistent and easy for children to use, successfully engaging them, so that they would like to play the game again and often. However, we are already working on the suggestions proposed by the children who participated in the system validation, so that the next update of the application will include more levels for each minigame with increased difficulty, as well as an online minigame mode. With these modifications, we expect to increase the children’s interest in the game, making the game even more capable of keeping children engaged.

## Figures and Tables

**Table 1:** Individual SUS scores and mean value (children without ADHD).

	SUS Score
User 1	92.5
User 2	90
User 3	82.5
User 4	77.5
User 5	85
User 6	92.5
User 7	90
User 8	82.5
User 9	90
User 10	92.5
Mean SUS score	87.5

**Table 2:** Individual SUS scores and mean value (children with ADHD).

	SUS Score
User 1	77.5
User 2	90
User 3	85
User 4	92.5
User 5	90
User 6	77.5
User 7	90
User 8	77.5
User 9	85
User 10	92.5
Mean SUS score	85.75

## Data Availability

The data and codes that support this study are available with the indentifier(s) at the link https://drive.google.com/file/d/1k8XEKIKGSavU1b2VqyGtBdNdN3jPgwl_/view?ts=60158417 (accessed on 4 October 2021).
